# Supplementary material for: New Insights into the Enhancement of Adventitious Root Formation Using N,N′-Bis(2,3-methylenedioxyphenyl)urea
Source: Plants (Basel). 2023 Oct 18;12(20):3610. doi: 10.3390/plants12203610 (PMC10610038; doi:10.3390/plants12203610)

Supplementary figures

Figure S1. Representative phenotypes of Arabidopsis pavement cells obtained under different treatments: A) hormone free (HF) as control condition; B) 1 μM 2,3-MDPU; C) 1 μM IBA; D) 1 μM IBA plus 1μM 2,3-MDPU. The pictures were taken 7 days after sowing by confocal laser scanning microscopy.

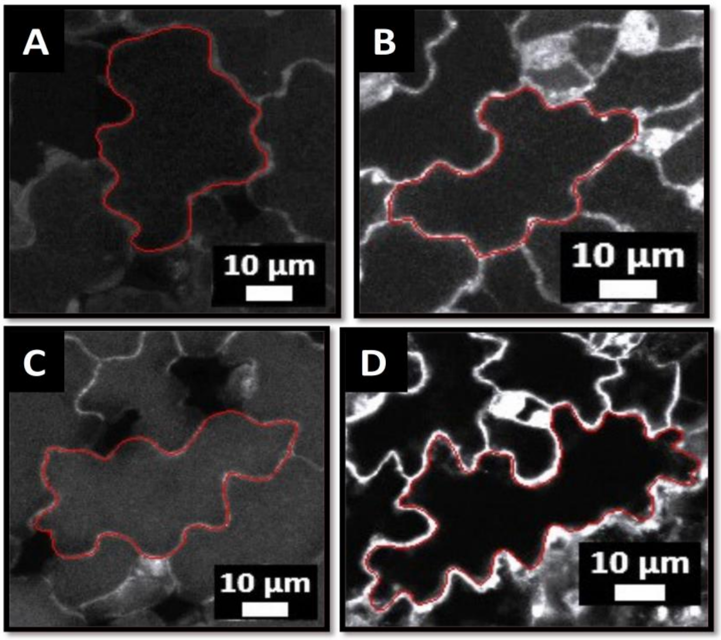

Figure S2. A) Dependence of the reaction rate of ZmCKX1 on trans-zeatin (tZ) concentration in the 0-100 µM range. The fitting of the data points (n=3 at each concentration) to the Michaelis-Menten equation (solid line) yielded a K_m of 6.8 µM. B) Lineweaver-Burk plots of the kinetics measured in the absence and presence of 2,3-MDPU at 5 and 10 µM concentration.

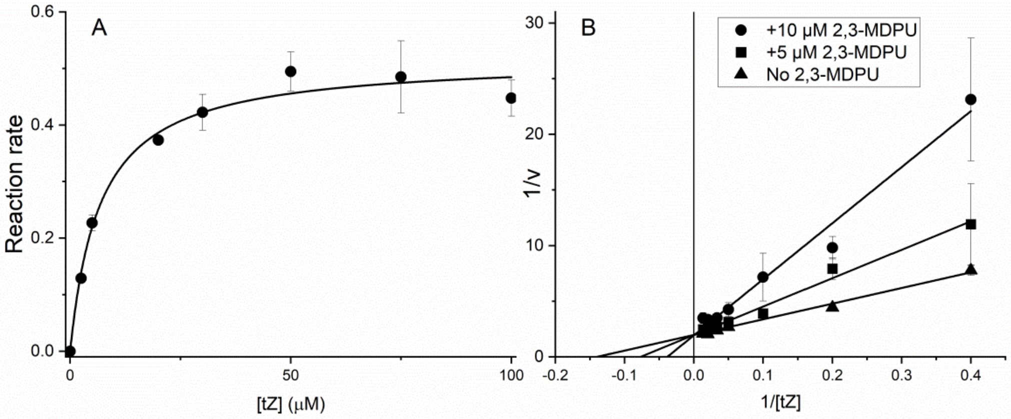

Supplement: Supplementary file 1 [file plants-12-03610-s001.zip › plants-2544914-supplementary.pdf]
